# Preliminary Findings on Low-Dose 1cp-LSD for Canine Anxiety: Exploring the Role of Owner Neuroticism and Psychopathology

**DOI:** 10.3390/vetsci12090872

**Published:** 2025-09-09

**Authors:** Elisa Hernández-Álvarez, Jaime Rojas-Hernández, Lucas F. Borkel, Domingo J. Quintana-Hernández, Tobías Fernández-Borkel, Luis Alberto Henríquez-Hernández

**Affiliations:** 1 Health Sciences Faculty, Universidad de Las Palmas de Gran Canaria, 35016 Las Palmas de Gran Canaria, Spain; elisa.hernandez106@alu.ulpgc.es (E.H.-Á.); jaime.rojas102@alu.ulpgc.es (J.R.-H.); lucas.fernandez101@alu.ulpgc.es (L.F.B.); 2 Asociación Científica Psicodélica, 35412 Arucas, Spain; domingoj.quintana@gmail.com (D.J.Q.-H.); tobiasfernandezborkel@gmail.com (T.F.-B.); 3 Asociación Canaria para el Desarrollo de la Salud a Través de la Atención, 35007 Las Palmas de Gran Canaria, Spain; 4 Faculty of Psychology, Universidad del Atlántico Medio, 35017 Las Palmas de Gran Canaria, Spain; 5 Center for MR Research, University Children’s Hospital Zurich, 8032 Zurich, Switzerland; 6 Unit of Toxicology, Clinical Science Department, Universidad de Las Palmas de Gran Canaria, 35016 Las Palmas de Gran Canaria, Spain

**Keywords:** animal welfare, anxiety, dogs, animal behavior, psychedelics, LSD, 1cp-LSD, neuroticism, human–animal bond

## Abstract

This study investigated the effects of low-dose 1cp-LSD, a prodrug of LSD, on canine anxiety, while exploring the influence of owner psychopathology on treatment outcomes. Seven dogs exhibiting anxiety-related behaviors were recruited and received 2.5, 5, or 10 µg of 1cp-LSD orally every three days for 30 days. One additional dog, which did not meet clinical criteria for anxiety but whose owner perceived it as anxious, received a placebo (saccharin) as an observational case. Anxiety levels were assessed using validated scales at baseline, post-treatment, and one month after treatment cessation. Owners’ neuroticism and psychopathological dimensions were evaluated using the IPIP and SA-45 scales. Linear regression analyses examined associations between anxiety reduction, administered dose, and owner psychological traits. A significant reduction in separation anxiety scores was observed post-treatment (mean: 11.3 ± 3.0, *p* = 0.023). Higher 1cp-LSD doses correlated with greater anxiety reduction (B = −1.15, 95% CI = [−2.29, −0.003], *p* = 0.050). Additionally, higher owner psychoticism scores were associated with smaller improvements in canine anxiety (*p* = 0.035). Anxiety levels remained low one month after treatment, with significant associations between canine anxiety and the owners’ hostility, interpersonal sensitivity, and paranoid ideation. These findings support the potential role of low-dose serotonergic psychedelics in managing canine anxiety and suggest an association between caregiver psychological traits and treatment outcomes. The sustained reduction in anxiety behaviors also suggests a meaningful and lasting improvement in animal welfare. However, significant results should be interpreted cautiously due to the small sample size. Further blinded, controlled studies are needed to validate these preliminary observations and assess broader applications in veterinary behavioral medicine.

## Simple Summary

This study explored whether small oral doses of 1cp-LSD, a legal prodrug of LSD, could help reduce anxiety in dogs. Seven anxious dogs were treated over 30 days, and their anxiety levels were evaluated before, after, and one month following treatment. An additional dog, whose anxiety was perceived by the owner but did not meet clinical criteria, received a placebo. This case was included for observational purposes only and was not part of the formal analysis. The results showed that dogs treated with higher doses of 1cp-LSD experienced greater reductions in anxiety, especially in separation-related behaviors. Notably, dogs whose owners scored higher on measures of hostility, interpersonal sensitivity, and paranoid ideation showed smaller reductions in anxiety. These findings suggest that low-dose psychedelics may have therapeutic potential for anxious pets, and that an owner’s emotional state can influence treatment success. Although the study involved a small number of animals, no adverse effects were observed, and improvements appeared to last beyond the treatment period. More research is needed, but this early work offers insight into new ways of supporting animal welfare through novel therapies.

## 1. Introduction

Anxiety is defined as a negative emotional state characterized by the anticipation of a threat, danger, or harm to the animal. It is, therefore, an adaptive response to a situation perceived as harmful by the animal [1], which in some cases may become excessive and disproportionate, leading to maladaptive outcomes [1]. It is an unpleasant and undesired state for the animal, which will seek to reduce or eliminate this emotional condition and develop anticipatory behaviors to prepare for potential events. Among the various types of canine anxiety, including generalized anxiety, aggression, and compulsive disorders, separation anxiety is the most common, with an estimated prevalence of 14–20% [2,3]. The three signs classically associated with separation anxiety in dogs are excessive vocalization (barking, whining, or howling), destructive behavior (chewing, scratching, or digging, often near exit points), and inappropriate elimination (urinating or defecating indoors), always in the owner’s absence [1]. Other signs of separation anxiety include excessive salivation, increased motor activity, continuous pacing, persistent panting, aggression when the owner leaves, stereotypic behaviors, vomiting, or anorexia [1]. Additionally, when the owner returns, a dog suffering from separation anxiety exhibits characteristic behaviors, including an excessively enthusiastic greeting, continuous physical contact, or persistent demands for petting and attention [4,5]. As a consequence, there is a significant deterioration in animal welfare, a weakening of the pet–owner bond and, in many cases, resulting in rehoming or even abandonment of the affected animals [6].

The treatment of separation anxiety is generally complex and lengthy, as it involves owner education, environmental modifications, and behavioral therapy for the animal [7]. In this context, the emotional bond between the animal and its owner becomes a key factor. Moreover, the emotional environment created by the people the dog lives with is perceived by the animal and can sometimes have a significant influence on its behavior [8,9]. It has been reported that owners’ personality traits have been significantly correlated with those of their dogs within the framework of the Five-Factor Model (FFM). This association may stem from a shared social environment and activities, facilitating emotional contagion, or from a selection process in which owners choose dogs that align with their personality and lifestyle [10]. Neuroticism is one of the five major dimensions in the FFM of personality and is associated with heightened sensitivity to stress and a lower threshold for emotional reactivity [11]. It is defined as a fundamental personality trait characterized by a tendency to experience negative emotions such as anxiety, depression, self-doubt, and emotional instability [11]. As a result, individuals high in neuroticism are more prone to experiencing psychological distress and interpreting situations as threatening or overwhelming. The association between owner neuroticism and canine behavior has been previously documented [12], indicating that dogs exhibiting aggression toward humans tend to be less sociable and are often owned by individuals who display lower emotional stability, greater detachment, and reduced tendencies for attachment-seeking behavior compared to owners of non-aggressive dogs [9]. This emotional interaction has been shown to affect treatment outcomes for canine anxiety and, in some cases, may be associated with the use of psychoactive drugs for management [13].

Maladaptive anxiety disrupts the neurochemical mechanisms that regulate an animal’s responses, justifying the use of specific drugs to restore normal brain chemistry [7]. This results in increased plasma levels of dopamine and serotonin [14], which are the primary targets of most pharmacological treatments for anxiety [15,16]. The pharmacological approach to anxiety includes the use of (i) antidepressants, such as selective serotonin reuptake inhibitors, serotonin antagonists, tricyclic antidepressants, and monoamine oxidase inhibitors; and (ii) anxiolytics, including benzodiazepines and azapirones, either as monotherapy or in combination [7,17]. However, these drugs exhibit highly variable success rates, require individualized dose adjustments, and are associated with undesirable effects such as sedation and cardiac complications [7,18]. Additionally, they can lead to tolerance and, in some cases, exacerbate anxiety symptoms [17]. In the development of new anxiety therapies, psychedelics have been shown to be useful not only in humans [19] but also in the context of canine anxiety [20,21]. The mechanism underlying this effect involves the modulation of serotonin levels [22]. Classic psychedelics, such as lysergic acid (LSD), exert their action through the modulation of serotonergic receptors, primarily the 5-HT2A receptor [23,24]. These compounds have been shown to be safe in terms of dosage, non-addictive, and free from severe pathophysiological adverse effects [25,26]. However, their use remains prohibited despite historical evidence supporting their efficacy in treating various mental disorders [27].

This study aimed to evaluate the potential anxiolytic effects of low doses of 1-cyclopropionyl-d-lysergic acid diethylamide (1cp-LSD), a legal LSD analog, in a cohort of dogs diagnosed with separation anxiety, following a structured treatment protocol as previously published [21]. The present work constitutes a direct follow-up of our earlier single-case study, in which preliminary observations suggested potential anxiolytic benefits of 1cp-LSD in canine anxiety [20,21]. Additionally, this study further explored the role of owner neuroticism and psychopathology as potential modulators of treatment outcome. We hypothesize that administration of low-dose 1cp-LSD will result in a reduction in canine anxiety, moderated by owner psychopathological traits. Owner assessments were conducted under blinded conditions to minimize expectation bias, although subjective reporting may still influence results. By expanding from a single subject to a prospective cohort design, the current study builds upon those initial findings and provides novel, systematic data that reinforce the rationale for further investigation.

## 2. Material and Methods

### 2.1. Study Design and Patient Recruitment

**Figure 1:**
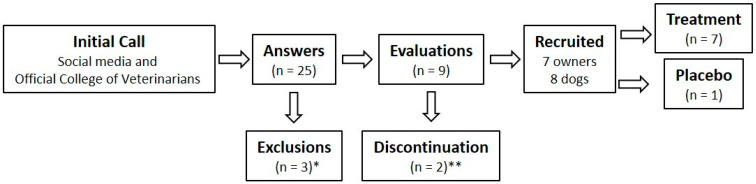
Flowchart illustrating the prospective recruitment of dogs and their owners. * One subject only wished to contribute to the project; one subject was unwilling to medicate their dog; and one subject opted for the employment of a canine trainer. ** Although the subjects completed the baseline anxiety assessments, they failed to respond to subsequent follow-up attempts.

This pilot study was designed to explore the feasibility and preliminary effects of low-dose 1cp-LSD administration in dogs with anxiety-related behaviors. Participant recruitment was conducted through social media announcements by the Psychedelic Scientific Association (https://www.asociacionpsicodelica.com/en Accessed on 15 October 2024) and via the email list of the Official College of Veterinarians of the Province of Las Palmas, Spain. The recruitment period was open from 15 October to 15 November 2024. Details of the prospective recruitment process are outlined in Figure 1. During this period, 25 individuals responded, believing their pet suffered from some form of anxiety. Three cases were initially excluded: one individual did not own a dog and only wished to collaborate, another declined pharmacological treatment for their dog, and a third opted for behavioral management with a trainer instead of participating in the study. The remaining respondents completed a baseline canine anxiety assessment to determine study eligibility. A total of nine owners completed this questionnaire and were subsequently invited to an in-person meeting where the study design was explained, and informed consent was obtained. Ultimately, seven owners and eight dogs were prospectively recruited, as one participant owned two dogs that met the inclusion criteria (Figure 1). Five out of seven participants lived in an apartment, and four out of seven lived alone. Regarding the dogs included in the study (*n* = 8, as both pets of participant 5 were enrolled), six were female and two were male. Mean age was 6.3 ± 3.9 years (range = 2–12). Four dogs were purebred, two were mixed breeds of known lineage, and the remaining two were crossbreeds of unknown origin. Half of the animals were neutered, and five out of eight cohabited with other animals. Three dogs came from a shelter, while the remaining five had been adopted (Table 1). Among the recruited subjects, one dog did not meet the inclusion criteria for clinical anxiety but was included as an observational placebo case, based on the owner’s belief that the animal was anxious. The owner was blinded to their pet’s anxiety levels. The first treatment was initiated on 17 November 2024. The last dog to begin treatment started on 20 January 2025. The study was concluded on 23 March 2025, during which time all animals were treated and evaluated at the various time points.

**Figure 2:**
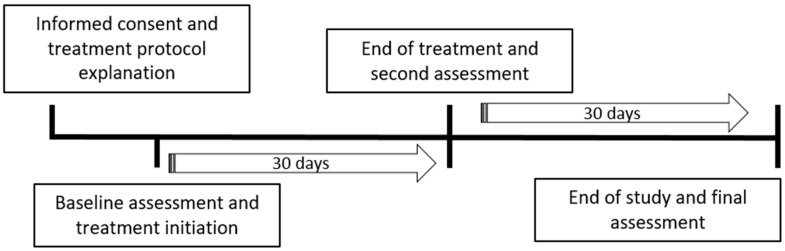
Longitudinal study design with a 60-day duration.

To ethically justify the administration of active treatment to all dogs meeting inclusion criteria, while minimizing expectation bias in owner reports, a blinded, authorized deception strategy was employed. Owners were informed that their dogs could be randomly assigned to either a treatment or placebo group; however, in practice, all dogs meeting clinical criteria for anxiety received 1cp-LSD, as withholding treatment was deemed ethically inappropriate in this pilot phase. One additional dog, which did not meet inclusion criteria, received placebo. This design allowed for blinding of owners to treatment status while ensuring that animals with clinical anxiety were not denied potentially beneficial intervention. The study followed a longitudinal design (Figure 2). After obtaining informed consent and completing the baseline assessments of canine anxiety and owner neuroticism, treatment was initiated. As in previous research [21], the medication was administered orally once every three days for 30 days (number of doses per animal = 10). At the end of the treatment phase, a second evaluation of canine anxiety and owner neuroticism was conducted. The study continued for an additional 30-day drug-free period, after which a final assessment of both variables was performed. Thus, the total study duration was 60 days (Figure 2).

This study adhered to the Three Rs (3Rs) principles for animal experimentation and was conducted in compliance with Directive 2010/63/EU of the European Parliament on the protection of animals used for scientific purposes. It also followed the recommendations of the National Research Council for the care and use of animals and was conducted with informed consent from all owners. While 1cp-LSD is not an approved veterinary medication, its use in this pilot study was ethically reviewed and approved by the Animal Experimentation Ethics Committee of the University of Las Palmas de Gran Canaria (Ref No. OEBA_ULPGC 02/2024). The decision was supported by the growing scientific interest in the potential anxiolytic properties of serotonergic psychedelics, the well-documented neurochemical similarities between canine and human anxiety, and the use of low, sub-perceptual (non-hallucinogenic) doses. Given the presence of clinically relevant anxiety symptoms, administering low-dose 1cp-LSD to eligible dogs was considered ethically preferable to withholding treatment. To reduce expectation bias, owners were blinded to treatment allocation.

### 2.2. Rationale for the Sample Size

Given the exploratory and pioneering nature of this study, the sample size was carefully considered in light of practical, ethical, and methodological constraints. Several important considerations have to be taken into account.

Ethical constraints: this is a prospective study in companion dogs living in a human–animal bond, using an experimental drug without veterinary marketing authorization. Both the potential impact on the animals and the implications for their owners impose strict ethical limitations on recruitment. In accordance with current legislation in Europe and Spain, these dogs do not qualify as experimental animals, as they were privately owned companion animals living in their home environment and participating in non-invasive procedures with their caregiver’s consent.

Homogeneity of subjects: the prospective nature of the study, involving privately owned dogs in a real-life context, inherently reflects the variability encountered in clinical practice, while maintaining a consistent baseline of health and age category across subjects. Nevertheless, it should be acknowledged that a high degree of between-subject variability, both in canine behavioral expression and in owner psychological traits, may have contributed to the observed outcomes. Although the longitudinal within-subject design helps to partially mitigate this effect, the small sample size prevents ruling out individual variability as a major factor influencing the findings.

Precedent in literature: previously published experimental pharmacological studies in dogs have employed small sample sizes [28,29,30,31]. This underscores that, in pioneering studies involving novel compounds, small cohorts are not only common but often necessary.

These considerations, together with the need to safeguard both animal welfare and the trust of their owners, strictly limited the number of subjects that could be enrolled.

### 2.3. Instruments for Measuring Canine Anxiety and Human Neuroticism and Psychopathology

To assess canine anxiety, two validated scales were used: a specific scale to determine the degree of separation anxiety (validated in [32]) and a second scale to assess the level of general anxiety (validated in [33]). The tool specifically developed to objectively assess canine separation anxiety comprises 17 questions scored on a scale starting from 0 and extending beyond 21 points. The scale assesses the main components of separation anxiety (vocalization, destructive behavior, inappropriate elimination, salivation, and motor activity) considering both intensity and frequency. The scoring interpretation is as follows: 0 to 3 points indicate the absence of separation anxiety, 4 to 8 points suggest a mild attachment disorder, 9 to 15 points correspond to moderate separation anxiety, 16 to 20 points reflect marked separation anxiety, and 21 or more points indicate severe separation anxiety [21,32]. To assess general anxiety, the LCAS (Lincoln Canine Anxiety Scale) was used [33]. This scale consists of 16 questions about anxious behavior, each rated from 0 to 5: running around; excessive salivation; hiding; destructive behavior; cowering/fearful behavior; restless/nervous; aggressive behavior; freezing behavior; barking/snorting/growling; panting; vomiting/defecation/urinating; constantly seeking the owner; hypervigilance and environmental scanning; escaping or running away; trembling/shaking; self-injurious behavior. A score of 0 indicates the absence of the anxious behavior, 1 suggests that the behavior is sporadic or of very low intensity, and 5 represents a highly frequent or intense anxious behavior. A total score of 30 points or more is considered indicative of generalized anxiety, as previously reported. Canine anxiety was assessed by the veterinary staff using validated instruments, based on structured information provided by the owners. This approach reflects standard practice in veterinary behavioral research, where owner input is essential for evaluating context-dependent emotional states that are not directly observable in clinical settings. To be included in the study and receive treatment, an animal had to exhibit anxiety-related behavior on both assessment scales.

The study protocol included the administration of psychometric scales to the owners, reviewed and approved by the Animal Experimentation Ethics Committee of the University of Las Palmas de Gran Canaria (Ref No. OEBA_ULPGC 02/2024). All owners received detailed information and signed an informed consent form explicitly covering their participation through the completion of the psychometric questionnaires. To assess the emotional stability (neuroticism) and psychopathological symptomatology of the pet owners included in the study, two previously validated scales were used: the International Personality Item Pool (IPIP) [34] and the Symptom Assessment-45 Questionnaire (SA-45) [35]. Regarding the IPIP scale, we specifically selected the emotional stability dimension, consisting of 10 items rated on a 1 to 5 scale, where 1 indicates strongly disagree and 5 indicates strongly agree. Assessed items included “I am calm most of the time”, “I get offended easily”, “I get upset easily”, “I frequently change my mood”, “I get angry easily”, “I have frequent mood swings”, “I get irritated easily”, “My emotions overwhelm me”, “I often feel sad”, and “I frequently complain about things”. The IPIP score was calculated as the mean of the responses to the 10 items, considering that the response value for item 1 was reversed, as it is formulated in the opposite direction compared to the other nine items [34]. For the assessment of psychopathological symptomatology, the SA-45 scale was used. This instrument consists of 45 questions rated on a scale from 0 to 4, where 0 indicates “not at all” and 4 indicates “extremely present.” The 45 items are grouped into nine dimensions, each comprising five specific items: depression, hostility, interpersonal sensitivity, somatization, anxiety, psychoticism, obsessive–compulsion, phobic anxiety, and paranoid ideation (Appendix A). The score for each dimension was calculated as the mean of the responses to the specific items within that dimension [35]. The assessment of the scales related to the owners was conducted by the psychologists of the research team.

### 2.4. Dosage of 1cp-LSD

1cp-LSD is a semi-synthetic psychedelic compound and an LSD analog, which has been available for research since July 2019 [36]. It undergoes hydrolysis by blood carboxylesterases, converting into LSD and functioning as a prodrug [36]. Through this process, it interacts with adrenergic and dopamine receptors and acts as an agonist of serotonin 5-HT receptors [23].

To ensure administration within the low-dose range and avoid intense psychedelic effects [37], all dogs were treated with approximately the same absolute dose of 1cp-LSD, using the available presentations of 2.5 µg, 5 µg, or 10 µg. Thus, the dosing scheme was not intended to be weight-adjusted but rather to guarantee exposure within a safe microdose range previously shown to be well tolerated and potentially anxiolytic in canines [20,21]. Given the absence of pharmacokinetic data for dogs, the human-equivalent dose (HED) was additionally calculated as an extrapolative approach to better estimate the effective exposure per subject. For this purpose, both body weight and body length were used to determine body surface area (BSA) following the Du Bois formula [38], normalized to a standard human of 80 kg and 180 cm:

BSA (m^2^) = 0.007184 × BW (Kg)^0.425^ × L (cm)^0.725^,

where BSA is defined as body surface area, BW as body weight, and L as length (nose-to-base of the tail). A ratio is then established between the body surface area of a human weighing 80 kg and measuring 180 cm in height, set at 1.99 m^2^, and the BSA of the dog. This ratio was multiplied by the administered dose to obtain the HED. The estimated values are shown in Table 2. This dosage has been demonstrated to be safe in human medicine [37,39,40] and has shown no adverse effects in canines [20,21]. Potential associations between body weight and the administered dose were examined. Neither Pearson’s nor Spearman’s correlation analyses revealed significant associations, indicating that body size did not confound the subsequent dose–response analysis.

The 1cp-LSD was legally obtained from an online supplier (AlphaChain B.V., Utrecht, The Netherlands). Each pill contained 10 μg of 1cp-LSD L-tartrate. Concentration of the substance was confirmed by the manufacturer using quantitative nuclear magnetic resonance spectroscopy. The compound was dosed and stored in individual containers, then administered orally by concealing it in a piece of ham and given to the animal at breakfast time according to the designated treatment protocol.

A saccharin pill was used as a placebo, administered once every three days to simulate the drug treatment while ensuring that the owner remained unaware of their pet’s assigned treatment group. The substance was obtained from a local market, with each pill weighing approximately 50 mg and containing around 12 mg of sodium saccharin. At this dose, saccharin is safe for the canine species [41].

The research team closely supervised the treatment’s progression, maintaining continuous communication with the owners throughout the study. No adverse effects were observed; however, the absence of adverse effects cannot be considered conclusive due to the small sample size.

### 2.5. Statistical Analysis

Descriptive analyses were conducted for all variables. Means and standard deviations were calculated for continuous variables. The normality of the data was tested using the Shapiro–Wilk test. In order to assess the effects of the treatment over time while accounting for interindividual variability, delta (Δ) values were calculated for each evaluated parameter. These increments represent the difference between scores at consecutive time points, allowing for a more precise comparison of changes relative to baseline values. This approach minimizes the influence of individual baseline differences and enhances the detection of treatment-related effects. The calculated Δ values were used in subsequent statistical analyses to determine the significance of changes in both canine anxiety and owner psychological status throughout the study. Comparisons between groups were performed using Student *t*-test. Bivariate correlations between continuous variables were tested using Pearson’s r. To explore which variables were associated with changes in canine anxiety scores, a linear regression model was applied, using the change in anxiety scores (Δ1 and Δ2) as the dependent variable. A series of univariate linear regression models were conducted to evaluate the potential influence of dose and each psychometric scale (IPIP and SA-45 dimensions) on anxiety score changes. Model assumptions were checked for normality and homoscedasticity. Only the seven dogs who met the inclusion criteria based on validated anxiety scales were included in the main statistical analyses of treatment efficacy. The dog excluded for low baseline anxiety was retained solely for exploratory observation of anxiety evolution during the untreated period and was not part of the inferential comparisons. Probability levels of <0.05 (two-tailed) were considered statistically significant. Given the exploratory nature and limited sample size of this pilot study, we complemented p-values with effect size estimates (Cohen’s d) to better interpret the magnitude of observed effects. Although some findings reached statistical significance, they should be considered clinically tentative and interpreted with caution due to the limited power and absence of confirmatory data. PASW Statistics (version 19.0, SPSS Inc., Chicago, IL, USA) was used to manage the database of the study and to perform statistical analyses.

## 3. Results

### 3.1. Descriptive Analysis of the Series

A total of seven owners and eight dogs participated in the study (Table 1). Among the participants, six were women and one was a man. The mean age of the owners was 40.7 ± 9.9 years (range = 27–53).

A total of seven dogs met the inclusion criteria and followed the treatment protocol. They received 2.5, 5, or 10 µg of 1cp-LSD orally once every three days for 30 days (10 doses in total), depending on their body weight (Table 2). The mean body weight of the study subjects was 8.8 ± 4.7 kg. To ensure that the animals remained within the low-dose range, the human-equivalent dose was calculated based on each participant’s body surface area. The mean 1cp-LSD dose per administration was 31.2 ± 5.8 µg. The dog that received the placebo (saccharin) did not meet the inclusion criteria, although its owner believed that the animal suffered from anxiety. The owner was a 53-year-old woman living with her husband and two children in an apartment. Her dog was a spayed mixed-breed female, weighing 5.5 kg, and had been adopted from a shelter (ID 7; Table 1 and Table 2).

The first dose of 1cp-LSD was administered on 17 November 2024, and the last dose on 19 February 2025. The study was officially completed on 20 March 2025.

### 3.2. Role of 1cp-LSD and Owner Psychological Profile in Canine Anxiety: Baseline to End of Treatment

The individual anxiety scores of the dogs and the psychoticism scores of their owners recorded before the start of treatment are provided in Table S1. For separation anxiety, the mean score was 21.4 ± 11.6 points (range = 8–40). The dog with the lowest score was ID 6, while the highest score was recorded for ID 4. Regarding the LCAS, the mean score was 40.4 ± 8.3 points (range = 30–53). The lowest score on this scale was recorded for ID 3, whereas ID 4 had the highest score. For the placebo-treated animal, the separation anxiety and LCAS scores were 6 and 17 points, respectively. Among owners, neuroticism, assessed using the IPIP scale, had a mean score of 1.8 ± 0.5 points (on a scale from 1 to 5). Among the dimensions evaluated with the SA-45 scale, anxiety had the highest score (mean = 1.3 on a scale from 0 to 4), while interpersonal sensitivity had the lowest (mean = 0.2 points). At the beginning of the treatment, the psychoticism level was 0.

After one month of 1cp-LSD treatment, canine anxiety and the psychological profile of the owners were reassessed, showing significant changes (Table 3). The separation anxiety score decreased to a mean of 11.3 ± 3.0, a statistically significant difference (*p* = 0.023). The LCAS-derived score was 29.9 ± 11.8 (Table 3). Although the pre–post change in LCAS scores did not reach statistical significance (*p* = 0.089), the effect size was large (Cohen’s d = 1.04), suggesting a potentially meaningful clinical impact. This discrepancy highlights the importance of considering effect sizes, especially in small exploratory studies where statistical power is limited. Notably, animal ID 4 improved from severe to moderate anxiety, with a reduction from 53 to 16 points on the LCAS (Table S1; Video 1 available at https://m.youtube.com/watch?v=wteb9jBszb8 accessed on 18 April 2025). Regarding the owners, a significant decrease in hostility and anxiety were observed (*p* = 0.017 and 0.047, respectively; Table 3). Meanwhile, the animal in the placebo group experienced an anecdotal increase in anxiety scores, particularly in the LCAS, which rose from 17 to 32 (Table S1), exceeding the cutoff value of 30.

**Figure 3:**
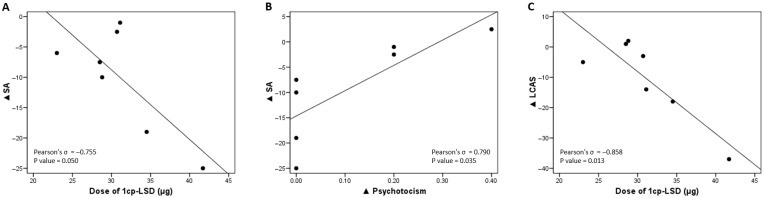
Significant bivariate correlations between the differences in anxiety scores (AS and LCAS) recorded after and before treatment, the administered dose (panels **A**,**C**), and the difference in owners’ psychoticism scores during the same period (panel **B**).

Delta (Δ) values were calculated for each evaluated parameter, representing the difference between scores at consecutive time points (post-treatment minus pre-treatment scores). The bivariate correlations showed significant associations between changes in canine anxiety scores and both the administered dose of 1cp-LSD and owners’ psychoticism scores (Figure 3). A negative correlation between the administered dose of 1cp-LSD and the change in separation anxiety (ΔSA) was observed, with higher doses being associated with greater reductions in anxiety (Pearson’s r = −0.755, *p* = 0.050; Figure 3A). No significant correlation was found between body weight and the administered dose, suggesting that the observed relationship between dose and ΔSA was not confounded by the dogs’ size. On the other hand, a positive correlation between ΔSA and changes in owners’ psychoticism scores was observed, suggesting that greater increases in psychoticism were associated with smaller reductions in separation anxiety (Pearson’s r = 0.790, *p* = 0.035; Figure 3B). Additionally, a strong negative correlation between the administered dose of 1cp-LSD and the change in LCAS scores (ΔLCAS) was detected, with higher doses leading to greater reductions in anxiety symptoms (Pearson’s r = −0.858, *p* = 0.013). Taken together, these findings suggest that 1cp-LSD may exert an anxiolytic effect in dogs in a dose-dependent manner, while also highlighting a potential moderating role in the owners’ psychological profiles in the treatment response. However, dose assignment was guided by body weight for safety rather than by pre-defined experimental groups; therefore, observed dose–response trends should be interpreted cautiously given the limited number of subjects at each dose level. The remaining bivariate correlations are provided in Table S2.

Finally, to explore which variables were associated with changes in canine anxiety scores, a linear regression model was applied (Table 4). Regarding separation anxiety, a significant negative association was found with the administered dose (B = −1.15, 95% CI = [−2.29, −0.003], *p* = 0.050), indicating that higher doses were linked to a greater reduction in anxiety scores. Additionally, an independent model showed a significant positive association between changes in owners’ psychoticism scores and changes in separation anxiety (B = 50.1, 95% CI = [5.37, 94.8], *p* = 0.035), suggesting that increased psychoticism in owners was associated with higher anxiety levels in dogs. For the Lincoln Canine Anxiety Scale (LCAS), the administered dose was also significantly associated with score reductions (B = −2.05, 95% CI = [−3.45, −0.64], *p* = 0.013), further supporting a dose-dependent anxiolytic effect of 1cp-LSD.

### 3.3. Role of Owner Psychological Profile in Canine Anxiety: End of Treatment to End of Study

The individual anxiety scores of the dogs and the psychoticism scores of their owners recorded at the end of treatment are provided in Table S1. For separation anxiety, the mean score was 10.7 ± 5.0 points (range = 3–17) at the end of the study (Table 3). The dog with the lowest score was ID 5, while the highest score was recorded for ID 8. Regarding the LCAS, the mean score was 23.3 ± 13.5 points (range = 3–39). The lowest score on this scale was recorded for ID 6, whereas ID 1 had the highest score. Of the owners, neuroticism, assessed using the IPIP scale, had a mean score of 1.9 ± 0.7 points (on a scale from 1 to 5). Among the dimensions evaluated with the SA-45 scale, obsessive–compulsion behavior had the highest score (mean = 1.28 on a scale from 0 to 4), while hostility had the lowest (mean = 0.06 points). No significant changes were observed in any variables when comparing the scores at the end of treatment and at the end of the study (Table 3). Compared to the pre-treatment scores, a significant decrease was observed in separation anxiety (*p* = 0.014), LCAS (*p* = 0.039), and paranoid ideation (*p* = 0.048).

Delta (Δ) values were calculated for each evaluated parameter, representing the difference between scores at consecutive time points (end of the study minus post-treatment scores). The bivariate correlations showed significant associations between changes in canine anxiety scores and owners’ psychoticism scores (Table S2). A positive correlation between ΔSA and the scores for Δ hostility, Δ interpersonal sensitivity and Δ paranoid ideation were observed (Pearson’s r = 0.707, *p* = 0.050; Pearson’s r = 0.844, *p* = 0.008; and Pearson’s r = 0.722, *p* = 0.043, respectively; Figure S1A), indicating that from the end of treatment to the end of the study, greater increases in these owner psychological traits were associated with higher separation anxiety scores in the dogs. Moreover, a positive correlation between ΔLCAS with Δ hostility and Δ interpersonal sensitivity scores were observed (Pearson’s r = 0.785, *p* = 0.021; and Pearson’s r = 0.816, *p* = 0.014, respectively; Figure S1B). These findings highlight the influence of owner’s personality and behavior on the dog’s anxiety. The remaining bivariate correlations are provided in Table S2.

Finally, to explore which variables were associated with changes in canine anxiety scores, a linear regression model was applied (Table 5). Regarding separation anxiety, a significant positive association was found with hostility (B = 4.77, 95% CI = [0.001, 9.54], *p* = 0.050), interpersonal sensitivity (B = 3.12, 95% CI = [1.14, 5.10], *p* = 0.008), and paranoid ideation (B = 3.38, 95% CI = [0.14, 6.61], *p* = 0.043). Regarding LCAS, a significant positive association was found with hostility (B = 20.4, 95% CI = [4.34, 36.6], *p* = 0.021), and interpersonal sensitivity (B = 11.6, 95% CI = [3.39, 19.8], *p* = 0.014). These findings support the notion that the human–animal bond and the owner’s emotional stability should be considered in relation to canine anxiety, particularly regarding hostility and interpersonal sensitivity. Moreover, the potential effect of 1cp-LSD treatment may be modulated by the owner’s emotional state.

## 4. Discussion

The present findings demonstrated that the administration of a low dose of 1cp-LSD, a legal prodrug of lysergic acid, given once every three days for one month, led to a reduction in anxiety scores in a cohort of dogs exhibiting this behavioral disorder. This study expands on a previous pilot trial conducted in a single patient, where similar outcomes were observed [20,21]. In the context of human psychedelic medicine, the efficacy of such treatments relies heavily on intention and integration processes, carried out in coordination with psychology specialists [39]. However, this approach is not feasible in veterinary medicine. While the therapeutic use of psychedelics in humans is increasingly supported by empirical findings, the precise mechanisms underlying their efficacy—particularly the role of subjective consciousness—remain under active investigation and conceptual debate [42]. In preclinical models, including rodents and zebrafish, anxiolytic outcomes are inconsistent, and the translational validity of these results is limited by the difficulty in interpreting complex behavioral states [43].

According to The Cambridge Declaration on Consciousness in Non-Human Animals, signed in July 2012, convergent evidence indicates that non-human animals possess the neuroanatomical, neurochemical, and neurophysiological substrates necessary for conscious states, as well as the capacity to exhibit intentional behaviors [44]. However, the presence of specific brain activity sufficient to support conscious experience does not necessarily imply that consciousness depends exclusively on such activity. While animal consciousness is widely accepted among neuroscientists, it remains debated whether all animals are conscious [45], and even more, the discussion extends to the existence of self-consciousness in animals [46]. The observations in the present study do not provide direct evidence of animal consciousness; rather, they offer a hypothesis derived from parallels with human research, acknowledging that the presence and nature of similar processes in non-human animals remain unclear. This framework allows for cautious interpretation of behavioral changes and for generating hypotheses to guide future studies in companion animals.

In general terms, consciousness can be defined in terms of qualitative feelings and refers to the subjective experience of perceiving environmental stimuli through the senses and developing a behavior conditioned to them. In other words, the experiential nature of the mind. The scientific community seems to agree that most animals have consciousness [46], something that, in the case of dogs, had been suggested since the classical conditioning experiments carried out by Pavlov [47]. However, the debate is wide in the fields of neuroscience, psychology, and philosophy, and there are different theories that attempt to explain the origin and functioning of consciousness in animals and humans [46]. The debate about animal self-consciousness is more complex, defined as a metacognitive awareness of one’s own mental states, awareness of one’s existence as a contiguous agent who moves through the world in time, or even as awareness of one’s self narrative [46]. Currently, there are three well-established sources of evidence for animal self-consciousness: mirror self-recognition, mental monitoring, and episodic memory [46], which serve as a conceptual framework, although none of these were assessed in the present study. Mental monitoring is defined as the ability to think about the accuracy of own thoughts, a capacity that appears to have been observed in rhesus macaques, dolphins, rats [48,49,50], and, recently, in canids [51]. Episodic memory is a sophisticated neurocognitive system that enables individuals to mentally travel through time, experiencing past, present, and future events subjectively. This “mental time travel” allows the “self”, through autonoetic awareness, to vividly recall personal, previously experienced events and to anticipate potential future experiences. Episodic memory builds upon and extends the capabilities of semantic memory [46]. This ability appears to be present in some animal species, including dogs [52].

**Figure 4:**
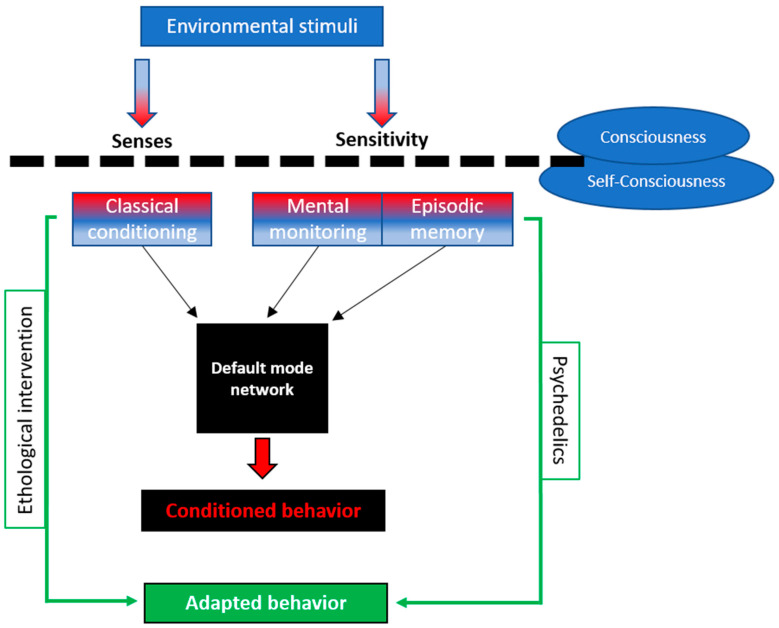
Conceptual model proposing a potential mechanism by which psychedelics may influence behavior through modulation of consciousness-related processes in dogs. Environmental stimuli are perceived through the animal’s senses and sensitivity, which contribute to its subjective experience and behavioral responses. Classical conditioning, mental monitoring, and episodic memory influence the animal’s behavior through the default mode network (DMN). Psychedelics, according to this model, could influence behavior by modulating DMN activity and altering consciousness-like processes. This, in turn, might lead to more adaptive responses to environmental challenges. Ethological intervention could be used to compare or control the effects of psychedelics.

While the pharmacological profile of classical psychedelics—such as their action as 5-HT2A agonists—is well-established, growing evidence suggests that this alone cannot explain their therapeutic effects. In fact, recent reviews point out the inconsistency of anxiolytic outcomes in standard animal models, including rodents and zebrafish, raising questions about the validity of such systems for psychedelic research [43]. In parallel, human studies consistently link psychedelic efficacy to subjective phenomena such as ego dissolution, insight, or altered perception of self, all of which implicate higher-order brain networks such as the default mode network (DMN). The default-mode network (DMN) is a set of functionally connected brain regions that play crucial roles in internal cognitive processing, implementing continuous evaluation and prediction of the environment to guide behavior [53]. Although with their particularities, dogs appear to possess a DMN [54]. Resting-state functional MRI studies in awake and lightly sedated dogs have revealed functionally connected networks involving anterior and posterior cingulate regions, consistent with the human DMN [54,55]. Group-level independent component analyses further identified distributed higher-order networks in dogs analogous to those in humans [56]. Complementary diffusion tensor imaging demonstrated structural connectivity between anterior and posterior cingulate regions, providing anatomical support for the functional dissociation observed in the canine DMN [57]. These findings suggest that dogs possess a DMN-like system, reinforcing their value as a comparative model for neural substrates of cognition and consciousness. In humans, psychedelics decrease DMN activity [58]. This leads to the disappearance of rumination and reduces activity related to self-identification and other higher cognitive and linguistic processes. Psychedelics generate altered states of consciousness not only based on the alteration of sensory perception [39]. While the anxiolytic effects observed can parsimoniously be attributed to serotonergic agonist of 5-HT receptors, as previously demonstrated for LSD and related compounds, our results also invite consideration of a complementary perspective. Specifically, if psychedelics modulate the canine DMN, they may provide indirect evidence of neurocognitive parallels with humans. This interpretation remains speculative, but underscores the potential of translational studies in dogs to generate testable hypotheses on the relationship between DMN dynamics, consciousness, and anxiety regulation. Given the lack of consistent anxiolytic effects in classical animal models and the central role of altered states of consciousness in human psychedelic therapy, it is necessary to consider alternative explanatory models that go beyond receptor pharmacology. To this end, Figure 4 presents a conceptual hypothesis of how psychedelics might act on animal consciousness and cognition, potentially through DMN modulation, thereby influencing behavior. While this model is speculative, it provides a scaffold for integrating ethological observations with contemporary theories in cognitive neuroscience and may inform future experimental design.

The present results have demonstrated that the behavior of dogs is associated with the mood of their owners. Importantly, while the dyadic relationship between owner and dog is a valid factor in understanding behavioral changes, the use of owner-based assessments introduces a significant limitation. Subjective perceptions, caregiver expectations, and their own psychological states may have influenced the reported outcomes, thereby limiting the ability to draw direct causal inferences about the intervention’s efficacy.

The relationship that humans establish with animals is sometimes determined by their psychopathological profile, such that it is possible to predict animal abuse in individuals with higher levels of psychopathy [59]. The affective relationship established between owners and their pets, although bidirectional in nature, exhibits an asymmetry in its proportionality [60,61]. This disparity leads to the formation of a bond that can act as a mechanism for emotional feedback, which, under certain circumstances, may result in the manifestation of animal behavioral problems. However, the regression analyses shown in the present study must be interpreted with caution due to the extremely limited sample size (n = 7), and the variability in both canine and human subjects. The magnitude of some coefficients (e.g., B = 50.1 for psychoticism) and wide confidence intervals are biologically implausible and likely reflect statistical noise rather than genuine associations. Thus, these findings should be interpreted with caution and seen as exploratory.

Dogs, as a species, exhibit a distinctive ethological repertoire, characterized by breed-specific intrinsic needs that are sometimes not adequately met. Insufficient provision of stimuli such as exercise frequency and intensity, or the quality of socialization with other individuals, both human and canine, can precipitate the onset of generalized anxiety and, in extreme cases, compromise animal welfare [62]. The behavioral development of dogs is well-studied, with the period between conception and sexual maturity of the animal being critical [63]. This period is known as early behavioral development and encompasses five phases, the most important of which is socialization, which takes place between the third and twelfth week of life. Anything to which the animal does not habituate or socialize during this period has the potential to provoke reactions of fear, flight, or aggression [63]. This is significant given the number of dogs that are adopted in life periods subsequent to these 3–12 weeks. Owners who lack knowledge of their pet’s specific socialization may face behavioral problems originating from incomplete, undesirable, or negative behavioral development. Once social maturity is reached, changes in behavior will occur if any of the following situations arise: (i) moderate/intense changes in group structure, (ii) moderate/intense changes in the animal’s environment, (iii) severe traumatic experiences, or (iv) medical problems affecting their behavior [63]. Socialization is a relevant factor that could potentially influence response to therapy. However, in the present study, most animals (IDs 1, 2, 3, 4, and 6) were adopted as adults, preventing assessment of the impact of early socialization by their current owners, while only two animals underwent this critical period with their present owners. Thus, the present experimental design does not allow evaluation of its effect on treatment outcome.

Concurrently, an increase in the intensity of attachment between owners and pets has been observed in recent decades, a phenomenon attributable to the evolution of prevailing human–animal cohabitation models in contemporary Western societies [64]. The type of relationship established between owners and their pets is determined by the demographic and psychological factors of the owners. For instance, women over 50 years of age with children under 18 in their care tend to form stronger emotional bonds with their pets compared to men, younger individuals, or those without children under 18 [65]. Interestingly, the only subject in the study who exhibited a worsening of anxiety scores belonged to this demographic profile. While this observation is anecdotal and not suitable for drawing conclusions, it does reflect findings from previous literature suggesting that dogs may be highly sensitive to the emotional states of their owners [66,67,68]. This association has been observed in the context of human anxiety, which can significantly modify animal behavior [69]. Rather than serving as evidence, this case serves as a reminder of the complex, bidirectional nature of owner–pet interactions and the potential for caregiver mental health to impact behavioral assessments in companion animals.

## 5. Strengths and Limitations

This study has several limitations that must be considered when interpreting the results. First, the authorized deception study design makes it difficult to disentangle the specific effects of 1cp-LSD from natural behavioral variability. Second, since owner psychopathological traits and canine anxiety behaviors were both assessed through owner reports, there is potential for same-source bias. Results may reflect owner psychology more than dog anxiety. Third, only eight subjects were included in the study, with high between-subject variability, limiting the generalizability of the findings and reducing statistical power. As such, some results—while failing to meet conventional thresholds for statistical significance—showed large effect sizes that may still reflect clinically relevant outcomes. This is particularly important in exploratory studies, where the distinction between “statistically significant” and “clinically tentative” must be interpreted with caution. The use of regression models with multiple predictors and a small sample may have introduced overfitting, despite the normal distribution of variables. In addition, regression models based on a small sample produce wide confidence intervals and implausibly large coefficients in some cases, suggesting a high degree of uncertainty and the possibility of statistical artifacts. Importantly, individual differences between dogs and their owners represent a major source of variability in the observed outcomes. While our longitudinal within-subject design helps to mitigate some of these effects, the preliminary nature of the study necessitates cautious interpretation and underscores the need for replication in larger, more homogeneous cohorts. As a consequence, these findings should be interpreted with caution and seen as exploratory and hypothesis-generating, requiring replication in adequately powered studies. Fourth, the placebo effect was not measured, meaning that owner expectations and observer bias may have influenced the reported outcomes. It should be noted that there is a complete lack of validated scales for assessing the placebo effect in the context of veterinary pharmacological studies. Fifth, while the study accounted for owner psychopathological traits, other environmental and contextual factors influencing canine anxiety were not controlled. Finally, the dose selection was based on human-equivalent dosing approximations due to the lack of validated interspecies conversion models for 1cp-LSD. While such methods provide a rough estimation, they do not account for species-specific differences in absorption, metabolism, or receptor sensitivity. This emphasizes the need for pharmacokinetic research to determine appropriate dosing regimens in canines. It is worth noting that breed-related differences may influence the pharmacokinetics and pharmacodynamics of the tested molecule, potentially affecting the variability of treatment responses observed in the study. Future research should address these limitations by employing blinded, placebo-controlled designs with larger cohorts and incorporating pharmacokinetic assessments to validate the therapeutic potential of 1cp-LSD in canine anxiety management. In addition, the use of objective behavioral assessments is required to better disentangle the complex relationship between dogs and their owners.

Conversely, this study presents several strengths that enhance its relevance and scientific contribution, offering preliminary evidence to inform and guide future rigorous trials. First, it is a pioneering investigation into the potential therapeutic effects of 1cp-LSD in canine anxiety, expanding upon previous single-case observations and reinforcing the feasibility of this approach. Second, the study employed validated anxiety assessment scales, ensuring objective and standardized measurements of behavioral changes. Additionally, to strengthen the interpretation of treatment effects beyond statistical significance, effect sizes (Cohen’s d) were calculated for each comparison. The Cohen’s d test quantifies effect size independently of sample size. This approach provides a more nuanced understanding of the magnitude of change, especially valuable in small-sample exploratory research. The Cohen’s d test has demonstrated that the results are of a magnitude not strongly limited by the sample size, further supporting the p-values obtained in the statistical analyses. The inclusion of owner psychological profiling allowed for an exploratory analysis of the interplay between caregiver traits and treatment outcomes, an often-overlooked factor in veterinary behavioral research. Third, the longitudinal design enabled the monitoring of both short- and medium-term effects, providing insight into the persistence of behavioral modifications after treatment cessation. Despite its small sample size, which is inherently limited by the study design and strong ethical considerations, the study offers valuable preliminary data, laying the groundwork for future controlled trials. Far from being a weakness, the number of animals included is comparable to those used in experimental studies of new drugs within the context of companion animals [28,29,30]. Lastly, the findings contribute to the broader discussion on animal consciousness and psychopharmacology, offering new perspectives on how serotonergic modulation influences compulsive and maladaptive behaviors in non-human species.

## 6. Conclusions

This study reinforces preliminary evidence that low-dose 1cp-LSD may reduce canine anxiety, with effects persisting one-month post-treatment, but these potential anxiolytic effects require further validation. The findings highlight the significant influence of owner psychopathology on treatment outcomes. Increases in hostility, interpersonal sensitivity, and paranoid ideation were associated with a less sustained reduction in canine anxiety after treatment cessation. These results emphasize the complex interplay between pharmacological intervention and the human–animal bond, suggesting that the owner’s emotional state may modulate the therapeutic effects of 1cp-LSD. However, owner traits may confound anxiety assessments, highlighting the need for blinded outcomes. Conclusions are limited by uncontrolled design and a small sample. Future studies should implement blinded, controlled trials with larger cohorts to further elucidate the potential of serotonergic psychedelics in veterinary behavioral medicine.

## Figures and Tables

**Table 1:** Characteristics of the dogs and their owners participating in the study.

	Owner Characteristics				Dog Characteristics						
ID	Gender	Age	Habitat	Household Size	Gender	Age	Breed	Neutering	Cohabitation Time	Other Animals	Past Details
1	Female	34	Country state	1	Female	2	Andalusian Rat Terrier	No	8 *	Yes	Adopted
2	Female	42	Apartment	2	Female	4	Yorkshire/Tekkel	Yes	2	Yes	Adopted
3	Female	47	Apartment	1	Male	4	Mixed	Yes	2.5	Yes	Shelter
4	Female	27	Apartment	1	Female	5	Pointer	No	1.5	No	Adopted
5	Female	32	Apartment	1	Female	3	Chihuahua	Yes	3	Yes **	Shelter
6					Female	12	Mixed (Rat Terrier)	No	3	Yes	Adopted
7	Female	53	Apartment	4	Female	8	Mixed	Yes	8	No	Shelter
8	Man	50	Rural house	2	Male	12	Shih tzu	No	12	No	Adopted

* Referred to months. ** IDs 5 and 6 belong to the same owner. Both dogs were recruited for the study. Shelter dogs were acquired directly from a shelter, whereas adopted dogs joined their owners via other routes, including unwanted litters, transfers from other rescues, or being found and rescued from the street.

**Table 2:** Experimental dose of 1cp-LSD administered to each participant, and its human-equivalent dose based on the canine body surface area calculation.

ID	Weight (kg)	Length (cm)	Dog BSA ^1^ (m^2^)	Ratio ^2^	Dose ^3^ (µg)	HED (µg)
1	8.2	61	0.35	5.8	5	28.8
2	11	70	0.43	4.6	5	23.0
3	7.5	65	0.35	5.7	5	28.5
4	18	60	0.48	4.2	10	41.7
5	2.7	41	0.16	12.3	2.5	30.7
6	7	60	0.32	6.2	5	31.1
7	5.5	73	0.33	6.0	Placebo	—
8	7.5	50	0.29	6.9	5	34.5

Abbreviations: BSA, body surface area; HED, human-equivalent dose. ^1^ Dog BSA was calculated according to the Du Bois formula. ^2^ The ratio was obtained by dividing 1.99 by the estimated canine body surface area, where 1.99 (m^2^) represents the theoretical body surface area of a 1.80 m tall, 80 kg human. ^3^ Oral dose administered to each participant in the study.

**Table 3:** Mean and range of the different scales used for dogs and their owners before treatment, after treatment, and at the end of the study (*n* = 7, excluded the subject that received the placebo).

	Pre-Treatment	Post-Treatment	End of Study	*p* Value *	Cohen’s d ^†^	*p* Value **	Cohen’s d ^††^
SA	21.4 (8–40)	11.3 (7–15)	10.7 (3–17)	**0.023 ^‡^**	1.20	0.578	0.14
LCAS	40.4 (30–53)	29.9 (16–42)	23.3 (3–39)	0.089	1.04	0.131	0.52
Neuroticism ^1^	1.80 (1.0–2.2)	1.9 (1.0–3.5)	1.9 (1.1–3.0)	0.235	−0.19	0.766	0.04
Depression ^2^	0.66 (0–1.4)	0.94 (0–3.4)	0.80 (0–1.8)	0.363	−0.28	0.582	0.14
Hostility ^2^	0.50 (0–1.0)	0.22 (0–0.6)	0.06 (0–0.2)	**0.017** ^‡^	0.74	0.248	0.50
Interpersonal sensitivity ^2^	0.20 (0–0.4)	0.93 (0–3.8)	0.60 (0–3.4)	0.250	−0.73	0.245	0.29
Somatization ^2^	1.10 (0–2.4)	0.71 (0–3.0)	1.17 (0.4–2.0)	0.154	0.38	0.241	−0.47
Anxiety ^2^	1.30 (0–2.4)	1.13 (0–3.6)	1.06 (0–2.2)	**0.047** ^‡^	0.15	0.779	0.07
Psychoticism ^2^	0	0.26 (0–1.4)	0.14 (0–1.0)	0.175	−0.34	0.103	0.15
Obsessive–compulsion ^2^	1.00 (0–2.8)	1.08 (0–3.4)	1.28 (0.2–2.6)	0.483	−0.08	0.412	−0.22
Phobia ^2^	0.30 (0–1.0)	0.51 (0–2.8)	0.23 (0–1.6)	0.175	−0.21	0.140	0.33
Paranoid ideation ^2^	0.50 (0–1.0)	0.60 (0–2.2)	0.43 (0–2.2)	0.259	−0.11	0.395	0.18

Abbreviations: SA, separation anxiety; LCAS, Lincoln Canine Anxiety Scale. * Student T-test (pre-vs. post-treatment); ** Student T-test (post-treatment vs. end of study). ^†^ Effect size (Cohen’s d) for pre- vs. post-treatment; ^††^ Effect size (Cohen’s d) for post-treatment vs. end of study. Values > 0.8 considered large. **^‡^** Statistically significant but clinically tentative. ^1^ Subscale derived from the International Personality Item Pool (IPIP) inventory. ^2^ Each of the 9 factors derived from the Symptom Assessment-45 Questionnaire.

**Table 4:** Linear regression models assessing the relationship between changes in separation anxiety and Lincoln Canine Anxiety Scale scores after treatment compared to baseline (∆), with administered dose (∆ Dose) and changes in owner psychoticism scores (∆ Psychoticism) as predictors. B values represent unstandardized coefficients, with 95% confidence intervals [CI] in square brackets.

	B	[CI 95%]	*p* Value
∆ Separation anxiety			
Intercept	25.6	[−10.6, 61.8]	0.128
∆ Dose ^1^	−1.15	[−2.29, −0.003]	0.050
Intercept	−14.6	[−22.9, −6.37]	0.006
∆ Psychoticism	50.1	[5.37, 94.8]	0.035
∆ Lincoln Canine Anxiety Scale			
Intercept	53.2	[8.72, 97.7]	0.028
∆ Dose ^1^	−2.05	[−3.45, −0.64]	0.013

^1^ Dose calculated with the Du Bois formula.

**Table 5:** Linear regression models assessing the relationship between changes in separation anxiety and Lincoln Canine Anxiety Scale scores at the end of the study compared to the end of treatment (∆), with changes in owner hostility, interpersonal sensitivity and paranoid ideation scores as predictors. B values represent unstandardized coefficients, with 95% confidence intervals [CI] in square brackets.

	B	[CI 95%]	*p* Value
∆ Separation anxiety			
Intercept	−0.29	[−1.71, 1.66]	0.968
∆ Hostility	4.77	[0.001, 9.54]	0.050
Intercept	0.58	[−0.84, 2.00]	0.354
∆ Interpersonal sensitivity	3.12	[1.14, 5.10]	0.008
Intercept	0.22	[−1.52, 1.96]	0.768
∆ Paranoid ideation	3.38	[0.14, 6.61]	0.043
∆ Lincoln Canine Anxiety Scale			
Intercept	2.33	[−9.64, 1.76]	0.141
∆ Hostility	20.4	[4.34, 36.6]	0.021
Intercept	−1.99	[−7.91, 3.92]	0.441
∆ Interpersonal sensitivity	11.6	[3.39, 19.8]	0.014

## Data Availability

Data are contained within the article and Supplementary Material. Further inquiries can be directed to the corresponding author.

## Supplementary Materials

The following supporting information can be downloaded at: https://www.mdpi.com/article/10.3390/vetsci12090872/s1, Figure S1: Significant bivariate correlations between the difference in anxiety scores (AS (panel A) and LCAS (panel B)) recorded at the end of study and at the end of treatment, with the difference in owners’ psychoticism scores during the same period; Table S1: Individual scores for each pet and owner on the scales used in the study, recorded before treatment, at the end of treatment, and at the end of the study; Table S2: Bivariate correlations of the difference in anxiety scores recorded after and before treatment with the administered dose and the difference in scores related to the psychological dimensions of the owners during the same period.

## Appendix A. Assessment of the Nine Dimensions Derived from the Symptom Assessment-45 Questionnaire (SA-45)

Depression: (9) feeling lonely, (10) feeling sad, (11) lack of interest in activities, (27) feeling hopeless about the future, and (42) feeling useless.

Hostility: (7) outbursts of anger, (34) experiencing aggressive impulses, (35) urge to destroy objects, (39) frequent arguments, and (43) shouting or throwing objects.

Interpersonal sensitivity: (14) feeling misunderstood, (15) perceiving rejection from others, (17) feeling inferior, (32) feeling uncomfortable around people, and (36) feeling self-conscious; somatization: (18) muscle pain, (23) sudden hot or cold sensations, (26) numbness or tingling, (29) muscle weakness, and (31) feeling of heaviness.

Anxiety: (6) sudden fear, (12) feeling nervous, (30) excessive worry or tension, (38) panic attacks, and (41) feeling restless.

Psychoticism: (1) believing that one’s thoughts are being controlled, (4) hearing voices that others do not, (13) believing others can read one’s mind, (33) experiencing intrusive thoughts, and (45) self-punishing thoughts.

Obsessive–compulsion: (16) needing to do things very slowly, (20) excessive checking, (21) difficulty making decisions, (25) blank mind, and (28) difficulty concentrating.

Phobic anxiety: (3) fear of open spaces, (8) fear of leaving home alone, (22) fear of traveling on public transportation, (24) avoiding places or activities, and (37) fear of crowds.

Paranoid ideation: (2) blaming others for personal problems, (5) distrust of people, (19) feeling that others are watching or talking about oneself, (40) feeling unrecognized for achievements, and (44) believing that others are trying to take advantage of oneself.
