# Supplementary figures and images for: Preliminary Findings on Low-Dose 1cp-LSD for Canine Anxiety: Exploring the Role of Owner Neuroticism and Psychopathology
Source: Vet Sci. 2025 Sep 9;12(9):872. doi: 10.3390/vetsci12090872 (PMC12474181; doi:10.3390/vetsci12090872)

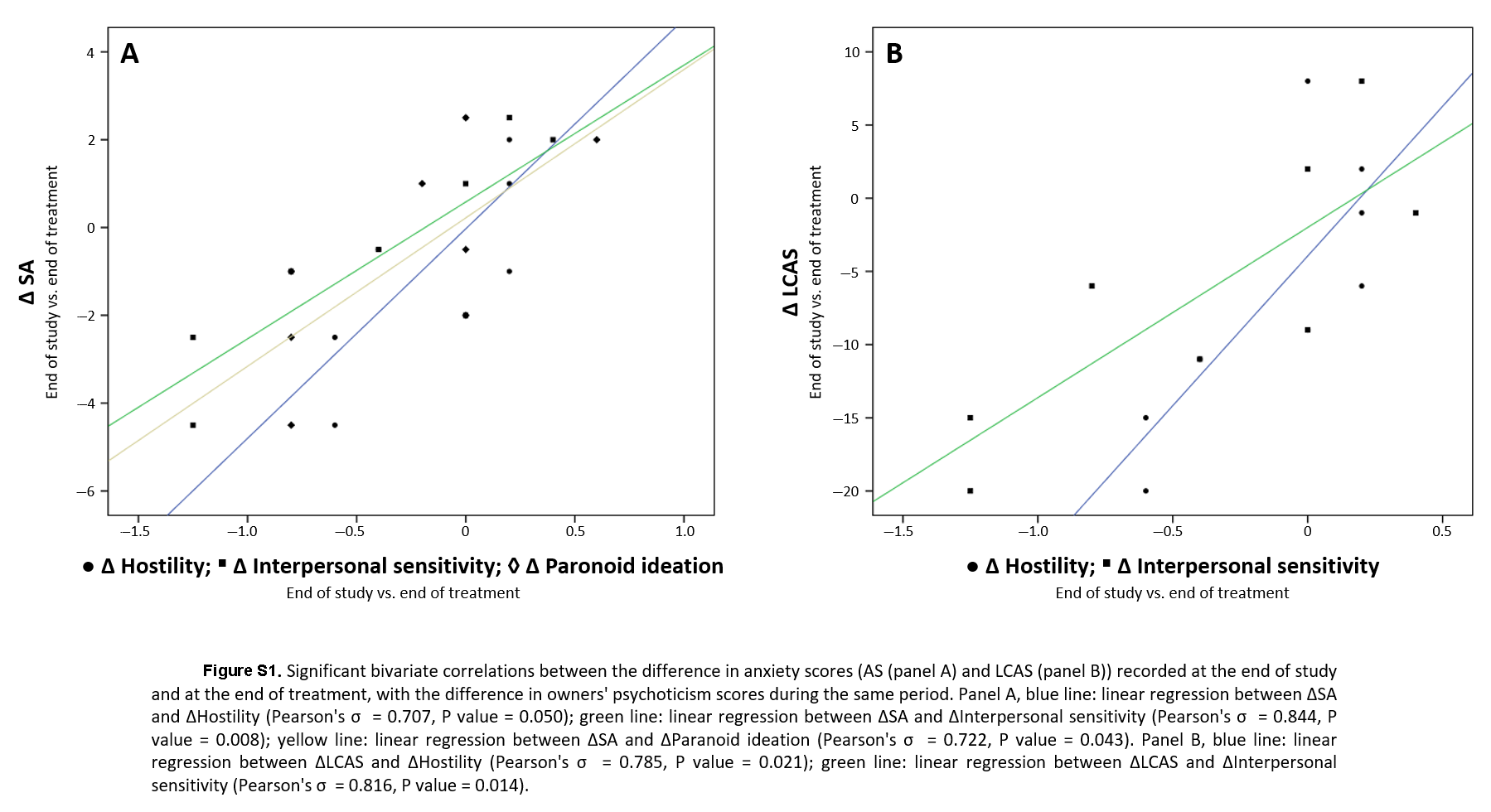

Supplement: Supplementary file 1 [file vetsci-12-00872-s001.zip › Figure S1.tif]
